# Omics as a Tool to Help Determine the Effectiveness of Supplements

**DOI:** 10.3390/nu14245305

**Published:** 2022-12-14

**Authors:** Anna Steg, Maria Oczkowicz, Grzegorz Smołucha

**Affiliations:** Department of Animal Molecular Biology, National Research Institute of Animal Production, ul. Krakowska 1, 32-083 Balice, Poland

**Keywords:** omics, dietary supplements, vitamins, plant extracts

## Abstract

There has been considerable interest in dietary supplements in the last two decades. Companies are releasing new specifics at an alarming pace, while dietary supplements are one of the less-studied substances released for public consumption. However, access to state-of-the-art and high-throughput techniques, such as the ones used in omics, make it possible to check the impact of a substance on human transcriptome or proteome and provide answers to whether its use is reasonable and beneficial. In this review, the main domains of omics are briefly introduced. The review focuses on the three most widely used omics techniques: NGS, LC-MS, NMR, and their usefulness in studying dietary supplements. Examples of studies are described for some of the most commonly supplemented substances, such as vitamins: D, E, A, and plant extracts: resveratrol, green tea, ginseng, and curcumin extract. Techniques used in omics have proven to be useful in studying dietary supplements. NGS techniques are helpful in identifying pathways that change upon supplementation and determining polymorphisms or conditions that qualify for the necessity of a given supplementation. LC-MS techniques are used to establish the serum content of supplemented a compound and its effects on metabolites. Both LC-MS and NMR help establish the actual composition of a compound, its primary and secondary metabolites, and its potential toxicity. Moreover, NMR techniques determine what conditions affect the effectiveness of supplementation.

## 1. Introduction

In the last 20 years, the interest and use of dietary supplements have significantly increased [1]. Consequently, much research has been conducted on the benefits of these substances, and a huge number of companies producing them have been created. However, there are many problems and challenges associated with dietary supplements, the first of which is the definition itself. The law defines dietary supplements in part as products taken by mouth that contain a “dietary ingredient”. Dietary ingredients include vitamins, minerals, amino acids, herbs or botanicals, and other substances that can be used to supplement the diet [2]. This is a broad and general definition. In addition, several other categories of products among different countries, such as natural health products (NHPs), complementary medicines, or food supplements, also fit this definition [3]. Another challenge is that each country, even among those with similar cultures, has different regulations regarding dietary supplements, and the same substance/mixture in one country can be labeled as a dietary supplement while in another it is considered a controlled substance. This makes it difficult for scientists to conduct consistent research and for consumers to choose an effective and high-quality product. 

Another problem is the existence of significantly different views about the testing and control of dietary supplements. It can roughly be distinguished between two extreme approaches. Some are in favor of treating supplements as drugs, which would allow for a thorough examination of effectiveness, side effects, and appropriate doses, while others believe that supplements should be considered individually and less restrictively, which is due to, among others, the fact that the effects of many substances are assumed only on the basis of traditional knowledge [4]. Undoubtedly, corporations that produce supplements benefit from a large discrepancy, conflicting opinions, and the lack of uniform regulations. It is estimated that the supplement market in 2021 was worth USD 151.9 billion [5], and manufacturers are releasing new products at an alarming rate, sparing no money for marketing purposes [6]. This means that pharmacies and drugstores are full of easily accessible dietary supplements whose exact composition and potential effects have not been studied [7,8,9,10,11]. Those concerns are especially true with plant-derived supplements, which are the fastest growing segment of the supplements industry and face the biggest problems in terms of quality control and standardization [4,12,13]. All this indicates a very great need for scientific collaboration to overcome the aforementioned international problems [14,15]. Many state-of-the-art methods allow for a thorough examination of the ingredient identity, adulterants, and positive and negative impact of a given supplement on the body’s functioning, determining the appropriate doses for specific social groups or interactions with other substances, as we wanted to demonstrate in this overview [16,17].

## 2. Fields Studying the Influence of Substances on Various Levels of Life Organization

Omics are the branches of science encompassing multiple molecular disciplines that aim to collectively characterize global sets of biological molecules, such as DNAs, RNAs, proteins, and metabolites [18]. Therefore, among omics, we can distinguish basic disciplines e.g., genomics, transcriptomics, proteomics, or metabolomics, and fields that intersect several approaches and examine interactions between different components such as nutritional genomics and microbiomics, pharmacogenomics, or foodomics.

### 2.1. Genomics

An entry point for other sciences is genomics, which is a field of science focusing on the collective characterization of a whole-genome of an organism, including interactions of genes with each other and with the environment. Genomics began to develop dynamically after the publication of the human genome sequence in 2001 [19,20]. Nowadays, genomics primarily uses modern, high-throughput DNA-sequencing techniques, microarrays, and bioinformatics. In the area of genomics, there can also be distinguished other omics disciplines such as transcriptomics (which studies the complete set of RNA transcripts that are produced at given circumstances in an organism or a specific tissue or cell) and epigenomics (which studies a complete set of epigenomics modifications on a gene expression) [21]. Genomic studies mostly make use of next-generation sequencing techniques.

### 2.2. Nutritional Genomics

Thanks to Garrod, who studied alkaptonuria, the concept of the influence of interactions between nutrition and genetics on phenotype was already known at the beginning of the 20th century [22]. However, the evolution of genomics contributed to the initiation of research on the influence of dietary components on gene expression, initiating a new field of omics—nutritional genomics—, which consists of two parts: nutrigenomics and nutrigenetics. The term nutrigenomics was first used in 2001 by Peregrin in order “to sum up the future of nutritional science into a single word” [23]. However, nowadays, this phrase is used to describe the study of interactions between dietary components and the genome plus the resulting changes in protein level, metabolism regulation and overall homeostasis [24]. In the case of nutrigenetics, its purpose is to check the body’s response to dietary ingredients concerning genetic differences [25]. The analysis of the relationship between diet and genes conducted within nutritional genomics allows for the identification of mechanisms in which nutrition components affect health and the development of civilization diseases [26]. The first publications on nutritional genomics appeared at the beginning of the 90s and mainly concerned obesity issues, including the dietary fats genes interactions. At that time, it was hoped that it would be possible to identify obvious genetic and nutritional factors that increase the predisposition to be overweight, and, as a result, cardiovascular diseases or diabetes. Indeed, recent analyses, suggest that more than ninety single genetic variants (usually SNP) are involved in body fatness, through pathways within the central nervous system (regulations of food intake) or within pathways of lipid metabolism or adipogenesis [19]. Moreover, studies showed that the intake of sugar-sweetened beverages and fried food interacts with the genetic risk of obesity [27,28]. 

Currently, more studies are being conducted to understand the mechanisms of the action of bioactive components on genes at the molecular level. Bioactive components of the diet are signaling molecules that can interact with one or more compounds, and as a result affect, for example, the process of gene expression in a quantitative and qualitative sense, lead to some changes in the body’s physiological response to nutrients [29]. Bioactive ingredients act on two levels, either as chromatin structure regulators or as direct regulators of the activity of nuclear receptors [30,31]. In nutrigenomic experiments, in vitro conditions are mainly used, such as model cancer cells, and recently, the so-called organoids are created, for example, from stem cells, but also experimental animals such as the Caenorhabditis elegans or mice [19,31]. It is becoming obvious that there are complex interactions between the microbiome, the immune system, and the whole-body metabolism and that dietary components may modulate many of these dependencies [32]. The uses of nutrigenomics are multiple. For instance, it can help with personalized medicine and personalized diets by assessing an individual’s nutritional requirements in order to prevent or treat obesity, diabetes, and metabolic disorder. Omics disciplines mainly employed in nutrition research are transcriptomics, proteomics, and metabolomics [33].

### 2.3. Proteomics

Proteomics is a broad and complex field of science that, on a large-scale, study protein properties, such as their function, structure, expression level, post-translational modifications or interactions, to obtain a global picture of cellular processes, networks, and disease processes [34]. Overall, the focus of proteomics is on the proteome, which is a portmanteau of the words protein and genome first used in 1995 by Marc Wilkins, and it refers to the totality of the proteins present in the cell line, tissue, or organism during the entire life cycle [35,36]. The study of the proteome is much more complicated than the study of the transcriptome or genome because the amount of protein in the body is influenced not only by mRNA expression but also by post-translational modifications or the current physiological state of the cell. The techniques conventionally used to study proteins are several types of chromatography for purification, enzyme-linked immunosorbent assay (ELISA) or western blotting for analysis, and gel-based approaches such as sodium dodecyl sulfate-polyacrylamide gel electrophoresis (SDS-PAGE) or two-dimensional gel electrophoresis (2-DE) for the separation of proteins. Nevertheless, when it comes to proteomics, high-throughput technologies such as protein microarrays, chips, mass spectrometry (MS) to analyze more complex protein mixtures, and X-ray crystallography or nuclear magnetic resonance (NMR) spectroscopy to provide a three-dimensional structure of the protein are more commonly used [37,38] These techniques provide a huge amount of data that require bioinformatic tools and databases to analyze, in order to predict the 2D and 3D structure of the tested proteins, interactions between them, and their response mechanism to various types of stresses, drugs, diseases, or dietary supplements.

### 2.4. Metabolomics

In the field of omics, we can also distinguish metabolomics, a dynamically developing area of science whose research goal is to identify and analyze the small endogenous and exogenous molecules (typically with sizes smaller than 1.5 kDa) called metabolites [39]. A complete set of substrates, intermediates, and products of metabolism in a cell, tissue, or organism is determined as a metabolome. Transcriptomic and proteomic analyses identify a comprehensive set of genes and their products being produced in the cell. Meanwhile, metabolomic studies can provide an insight into the current physiology of the cell [40]. In the late 1940s, Roger Williams’ research team developed the concept that every human being has his own “metabolic pattern” reflected by various components in body fluids components. This scientist, using paper chromatography, tested the presence of certain metabolites in urine and saliva, concluding that although the composition of these fluids varies from day to day and is distinctive from individual to individual, it is at the same time unique and characteristic to a given individual [41]. He conducted his research on samples taken from people suffering from, among others, alcoholism or schizophrenia and those staying in psychiatric hospitals, and he argued that each of these groups of people has a characteristic metabolic profile. Obviously, his research was mostly qualitative in nature. The real boom in the field of metabolomics took place about two decades later when more advanced techniques allowed for the quantitative description of tested samples [42]. Then, in 1971, Horning introduced the concept of the “metabolic profile”, after Dalgiesh proved that, by using gas chromatography-mass spectrometry (GC-MS), it is possible to measure the levels of components in physiological fluids and tissue extracts [43,44]. At the same time, NMR spectroscopy also gained importance in the study of the metabolome, thanks to Seeley, who in 1974 presented the usefulness of this technique to study metabolites in human tissues, proving, the basis of muscle that almost all ATP molecules are complexed with magnesium [45]. As with the genome or the proteome, the human metabolome was also examined in 2007 using previously mentioned variations of techniques—NMR and MS. The current version of The Human Metabolome Database (HMDB) contains information on 2280 drug metabolites, 25,000 human metabolic and disease pathways, and 28,000 food components and food additives [46].

### 2.5. Foodomics

The last category of omics discussed in this review is foodomics. It is a term first introduced in 2009 at the Cesena conference and is defined as a discipline that studies food and nutrients through the use and integration of advanced omics techniques. Such research aims to provide a more holistic understanding of the interactions between food and the functioning of the organisms and, at the same time, aims to take care of the quality and safety of food of both plant and animal origin, in order to improve consumer’s well-being and health [47,48]. Foodomics is the comprehensive approach for the exploitation of food science in light of an improvement of human nutrition; in this context, previously described nutrigenomics is considered a part of the more general term—foodomics [47]. Foodomics includes four sections of omics: genomics, transcriptomics, proteomics, and metabolomics. This field requires a combination of food chemistry, biological sciences, and high throughput analysis, which is why its research and development is still quite limited [49]. Foodomics techniques are useful from the production to the consumption of food, starting with analyses of raw material—(its microbiological and biological safety), through assessing the impact of food processing, to the quality and safety control in distribution and production. For instance, foodomics tools can detect possible allergens or foodborne pathogens in the product and in the case of side-effects caused by food, can identify causative agents or biomarkers, such as peptides or metabolites, that are relevant for tracking microbial infections or food allergies [48,50].

## 3. Molecular Techniques Most often Used in Omic Studies

In nutrigenomic research, it is necessary to precisely determine the influence of the tested substances on biological processes in the organism at the following levels: transcriptome, genome, proteome, and metabolome. Advanced, high-throughput techniques that generate a massive amount of data must be used to obtain a holistic view of a given subject. The most commonly used omic techniques are briefly described below.

### 3.1. NGS

It took over 12 years of hundreds of scientists’ work and cost almost USD 3 billion to obtain the sequence of the human genome [51].

The Human Genome Project relied on a first-generation sequencing technique called the Sanger technique, and although it allowed for the achievement of groundbreaking results, by the end of the project in 2002, it was known that a much more efficient, large-scale, and less expensive technique was needed, in order for these efforts to contribute to the development of genomic personalized medicine accessible for millions of patients [52]. Several years later, NGS (next-generation sequencing) techniques are in use, that overcome the limitation of Sanger sequencing methods and allow the entire genome to be sequenced in one day for about USD 1000 [53]. NGS-based tests rely on identifying the differences between the genome of the test sample and the reference genome. These differences may arrive from changes to the DNA sequence, e.g., single-nucleotide polymorphisms (SNPs), or large (the whole gene) deletions/duplications [52]. There are two major categories of NGS techniques, sequencing by hybridization and sequencing by synthesis (SBS), while the second approach is the predominant one [54]; therefore, the following descriptions focus on this method.

The basic NGS process involves three steps: library preparation which involves fragmenting DNA/RNA into multiple pieces and adding adapters (oligonucleotides of known sequence) at each end of the template fragments; then, sequencing of the library; and the last step is data analysis.

The vast majority of the sequencing data are generated using Illumina technology, where fragments of DNA with ligated adaptors at the ends, are hybridized to the flow cell surface and then amplified into a clonal cluster through bridge amplification cycles. Proprietary modified and fluorescently labeled nucleotides are incorporated and identified directly by fluorophore excitation during synthesis reactions. The process is repeated for at least 300 rounds. As all four reversible terminator dNTPs are present during each cycle, natural competition reduces incorporation bias and raw error rates. NGS platforms allow research of the genome, transcriptome, or epigenome of any organisms, with the use of a wide variety of methods such as whole-genome sequencing, de novo sequencing, targeted sequencing, total and mRNA sequencing, methylation sequencing or CHiP sequencing (chromatin immunoprecipitation sequencing) [55]. SBS methods rely on much shorter reads (up to 300–500 bases) and have an intrinsically higher error rate than Sanger sequencing. Another limitation of this approach is the reliance on high sequence coverage to obtain an accurate sequence [54]. 

Although typical, bulk RNA sequencing (RNA-seq) is extremely useful in studying gene expression, gene variants, alternative splicing, etc., and it illustrates an average of numerous cell transcriptomes present in the sample, disregarding the differences between individual cells. Therefore, shortly after introducing high-throughput RNA-seq, a technique for performing single-cell RNA-seq (scRNA-seq) emerged. This approach first requires tissue dissociation and then the isolation of single cells using fluorescence-activated cell sorting (FACS) or microfluidics-based techniques, or mechanical micromanipulation. The individual cells are lysed and converted into cDNA, which is the amplifier and is used to create RNA-seq libraries. scRNA-seq is successfully used in several fields, helping to study cancer heterogeneity and its microenvironment, immunology, neuroscience, and developmental biology [56]. It can also be useful in establishing the effect of a given factor on a particular type of cells, for instance, neurons or immune cells.

NGS techniques contributed to the rapid development and are now leading methods in omics fields such as genomics, transcriptomics, metagenomics, and nutritional genomics. NGS techniques can be used to determine human’ or animal’ genomes and can also be useful for both the qualitative and quantitative assessment and the identification of included in supplements species of, for instance, herbs. NGS techniques can also reveal a diverse community of fungi that are associated with live plant material [57].

### 3.2. LC/MS

Sometimes, a combination of several seemingly different techniques allows the discovery of their new possibilities and usefulness in many scientific fields. An example of such a successful combination is the LC-MS method, which combines the physical separation capabilities of liquid chromatography (LC) with the mass analysis capabilities of mass spectroscopy (MS). The coupling of chromatography and mass spectroscopy has been a subject of interest for over 60 years. The first one, reported in 1958 was a combination of gas chromatography (GC) with MS [58]. In GC, the analytes are eluted from the separation column as a gas and can be directly electrically (EI) or chemically (CI) ionized in order to produce mass spectra. This is not possible in the case of liquid chromatography; thus coupling LC with MS was technically a much more significant challenge, and hence it was not commercially available until the 1970s [58,59].

Nowadays, besides liquid chromatography and mass spectrometry devices, the LC-MS system also includes an interface based on atmospheric pressure ionization (API) strategies, that is used to transfer components from the LC column to the MS ion source. Therefore, the sample is pumped through the high-performance liquid chromatography (HPLC) column, where analytes move through at different migration rates. This step separates mixtures with multiple components such as biological fluids, drugs, food, or pesticides. Then, the eluent is directed to MS, where mass determines the mass-to-charge ratio of ions. These data can be used to determine the exact molecular mass that helps to establish the exact molecular mass and structural information about the components of such samples. 

The LC-MS offers high selectivity, resolution, precise mass, and specificity compared to other chromatography techniques. However, at the same time, it is also expensive in terms of capital and running costs, and is high maintenance.

This method is used successfully in a variety of fields, for instance in proteomic or metabolomic studies for peptide mass fingerprinting, the metabolite profiling of human/animal tissue, and for the analysis of natural products or secondary metabolites in plants [60,61].

### 3.3. NMR

At the end of World War II, the nuclear magnetic resonance (NMR) phenomenon was discovered independently by two groups of scientists Felix Bloch and Edward Purcell. It began to be tested within just a few years, mainly in chemistry, leading to the observation that different compounds give different signals [62]. NMR is a physical event that occurs in all nuclei that contain an odd number of protons and/or neutrons (in other words: nonzero nuclear spin) (most frequently used are ^1^H and ^13^C), and it means that at a characteristic and specific resonance frequency it comes to the absorption and re-emission of electromagnetic radiation [63]. 

Nowadays, NMR spectroscopy is a powerful tool that can provide detailed and quantitative physical, chemical, electronic and structural information about molecules in solutions and in the solid state. Many varieties of NMR techniques have been developed, which are used in various fields, e.g., in medicine, in which the so-called magnetic resonance imaging (MRI) is used for cancer diagnosis, and in chemistry, where proton NMR is used to identify the constituent parts of compounds. Furthermore, NMR is also a leading technique in proteomics and metabolomics to obtain information from biological fluids about the state of the disease or the level of toxins, as well as in foodomics to measure, for example, the ratio between water and fat and a given food product [64]. However, it should be mentioned that the disadvantage of this method is its low sensitivity, which means that it can only be used for the detection and measurement of metabolites in relatively high concentrations [65].

## 4. Some Examples of Omics Studies on the Most Popular Dietary Supplements

Currently, dietary supplements are composed of various ingredients such as minerals, amino acids, fatty acids, fibre, plant extracts, prebiotics, probiotics, and adaptogens (metabolic regulators (of a natural origin) that were shown to increase the ability of the organism to adapt to environmental factors and to avoid damage from such factors [66]). However, the most common supplements are still those containing vitamins. On the one hand, it is understandable because the deficiency of any of the elements necessary for the proper functioning of our body can be dangerous, which in itself is an effective advertisement encouraging potential recipients to buy such preparations. On the other hand, the vast majority of vitamin deficiencies in the body are due to improper nutrition and can be relatively quickly “repaired” through a healthy and varied diet, without supplementation. Taking dietary supplements containing vitamins should always be consulted with a doctor and based on laboratory tests confirming the level of deficiency and the preparation’s composition and safety should be carefully checked. Due to the fact that dietary supplements do not have to be examined and controlled as thoroughly as in the case of drugs, the composition declared by the manufacturer may be inconsistent with the facts [10]. 

Moreover, multivitamin preparations may also contain substances that, taken at the same time, block mutual absorption or even cause side effects. In addition, even though, it is possible to define reference ranges for the level of a given vitamin for a specific group of people, as well as to indicate disease entities that may in some way be associated with a disturbed level of one of the vitamins, the impact of supplementation on health is not fully explored. All this indicates a great need for effective tools for the large-scale testing of the composition of supplements and checking the impact of a given preparation on metabolism or human transcripts. With the emergence of techniques such as NGS, LC/MS, and NMR, great hopes were raised for using them in order to understand the mechanisms of action and effectiveness of various supplements at different levels of the cellular organization-transcripts, proteome, and metabolome. The proof here is the significant number of works created since 2005 concerning attempts to use these techniques.

The following is a brief review of some of the studies that used various omic techniques to investigate the composition or health effects of various groups of supplemented substances, such as vitamins and plant extracts. As the number of different categories and ingredients of dietary supplements is enormous, and there are plenty of studies on each of them, we decided to show only a fraction of them. We selected supplements and research examples that well illustrate the use of various omic techniques in the study of supplements. We wanted to show that the use of various omic techniques on different substances gives a wide range of results that for the study of both the supplemented product itself and the effect of its supplementation on the body. We paid special attention to vitamin D, because it is one of the most frequently supplemented vitamins, on which there are many new scientific reports and around which there are still lots of controversies, primarily regarding the doses, forms of supplementation, and potential impact on the functioning of the body [67]. The second group of compounds, apart from vitamins, are plant extracts. This choice is mainly because it is the fastest-growing branch of the supplements market, but also herbal dietary supplements are the greatest challenge to study. One of the reasons for this is because most of the premises about the effect of a given plant ingredient is based on traditional beliefs, and there are no data on the toxicity of these products. Moreover, there is a large genetic variability between different subspecies and varieties, and active ingredients obtained from plants are often sensitive to standard methods of purification, detoxification and disinfection, which means that these products are often with traces of pesticides or microbes [4,12].

### 4.1. Vitamins

#### 4.1.1. Vitamin D

The main focus of the review is research on vitamin D due to the fact that, of all the supplements, vitamin D supplementation is the most popular one (according to The ConsumerLab survey Dietary Supplement Consumer Trends and Preferences Report (2020) [68]. In fact, it is the only vitamin whose supplementation is widely recommended, especially in the fall and winter. The reason is that many population studies have shown vitamin D deficiency in most of the respondents. The shortage of this vitamin was intrinsically linked to rickets. Nowadays, all the current recommendations are based on bone health [67]. Unfortunately, the recommended daily doses vary significantly depending on the continent and even the country. The European Food Safety Authority recommends an intake of 600 IU (15 μg)/day in healthy adults, with a maximum recommended intake of 4000 IU/day, while the UK Scientific Advisory Committee on Nutrition recommends the intake of 400 IU/day for all [67]. Available studies, mostly RCTs (randomized controlled trials), suggest beneficial effects of vitamin D supplementation on bone mineral density and bone mineral content (BMD/BMC) with doses of about 10–25 μg/day (400–1000 IU), with the indication that the effects may depend on calcium intake [69]. There were also some studies suggesting that vitamin D supplementation affects muscle strength/function, fracture risk, or overall immune system functioning, but these require further research. 

The most frequently used indicator of vitamin-D status is a quantification of 25(OH)D in serum, for which the half-life is about 2–3 weeks [70]. The parent vitamin D in plasma has a much shorter half-life (0.5–5 days), which limited efforts to overcome technical challenges in its measurement [71]. One study showed that vitamin D concentration in serum may respond linearly to the dose of vitamin D administered daily, while in contrast to that, the concentration of serum 25(OH)D appeared to be steeper at low doses and approached a plateau as the dose of supplemented vitamin D increased [70,72]. Historically, serum vitamin D concentration was determined by competitive protein binding assay (CPBA) or HPLC [73,74]. These methods have limitations, such as low specificity in the case of CPBA, or limited throughput [75]. MS is the preferred analytical method for the quantitation of small molecules, such as vitamin D, because it offers high analytical sensitivity, specificity, and reproducibility. At present, LC-MS/MS assays are the preferred and routine method for measurements of 25(OH)D concentration, but there are only a few studies describing the use of LC-MS in measuring circulating vitamin D [76]. Based on the listed above reports, Best et al. from the University of Washington assumed that the serum vitamin D concentration may be a valuable biomarker that, opposite to 25(OH)D concentrations, proportionally reflects vitamin D uptake. Therefore, they made an attempt to optimize the LC-MS/MS method for this assay [71]. The group conducted two pilot studies where people were assigned into groups with different doses of vitamin D supplementation. Blood and SUBQ (subcutaneous) adipose tissue were collected at baseline and after 3 months of supplementation. With the use of the LC-MS/MS technique, the vitamin D3 and 25(OH)D3 levels were quantified. One of the findings was that with supplementation, the serum vitamin D3 concentration increased proportionally to the dose and reached a plateau by 1 month of treatment. Serum and adipose tissue vitamin D3 concentrations were correlated, and the dose-response of vitamin D3 in adipose mirrored that in serum. By contrast, the 25(OH)D3 response to supplementation was less than proportional to the dose and reached a plateau after 3 months, which is consistent with some meta-analyses that determined the that dose-response of serum 25(OH)D concentration to vitamin D intake was nonlinear. Therefore, the optimization and application of a modern LC-MS/MS method demonstrated the potential importance of the serum vitamin D concentration as a biomarker of actual vitamin D exposure in response to supplementation, in contrast to the currently used measurement of the 25(OH)D3 level.

Many reports indicate a link between vitamin D deficiency and an increased risk of developing a number of diseases, including civilization diseases or cancer. Even though the impact of vitamin D supplementation on gene expression has been extensively studied, much is still unknown [77,78,79,80,81,82,83]. This is where modern methods, such as NGS, can be helpful, as shown in studies conducted by Silva et al. [84] that analyzed the global gene expression of the human-derived Caco-2 cell line treated with vitamin D3. The results showed that genes involved in neuropeptide signaling, inflammation, cell adhesion, and morphogenesis were differentially expressed. The most considerable impact of vitamin D could be seen in the case of genes implicated in zinc, manganese, and iron homeostasis, such as ceruloplasmin, haptoglobin, and ZnT10 coding genes. These results may suggest that vitamin D3 stimulates the release of zinc and manganese into circulation to reach organs in need, for instance, to stimulate bone formation, preventing osteoporosis. These findings require further investigation, but it can be seen that the use of the NGS method allows for setting new, previously unknown effects of supplementation and further research goals.

However, the effectiveness of a given dietary supplement often depends not only on the form and dose but also on the individual characteristics of the person taking this compound. The VitDbol study (NCT02063334, ClinicalTrials.gov) showed that the responsiveness of the participants to the vitamin D oral supplementation, which was measured in 25(OH)D_3_ and 1.25(OH)_2_D_3_ serum concentrations, was dependent on individual changes in PTH (parathormone) levels or chromatin accessibility at particular loci. Thanks to the use of FAIRE-qPCR (Formaldehyde-Assisted Isolation of Regulatory Elements) technology on isolated PMBCs (peripheral blood mononuclear cells), participants were segregated into high, mid, or low responders [85]. As shown in the same intervention trial, although taking a given supplement resulted in a relatively small increase in the 25(OH)D_3_ serum concentration, this was found to be sufficient to induce significant changes at multiple sites in the epigenome of human leukocytes, as shown by assessing epigenome-wide chromatin accessibility by applying FAIRE-seq [86]. Moreover, it was shown that the effectiveness of vitamin D supplementation may be associated with BMI (body mass index) and serum fructose. These conclusions were reached by McClorry et al., who by examining the serum metabolome (assessed from children supplemented with vitamin D3) using the 1H-NMR method, showed that changes in S-25(OH)D concentrations were negatively correlated with the change in fructose concentrations [87]. 

Despite the similar exposure to sunlight, the same latitude and lifestyle, and similar diet, gender, and age, it turned out that in the group of people there were significant differences in vitamin D3 levels. One of the reasons for this may be genetic polymorphisms, as exemplified in the Filipino adult population, where with the use of targeted NGS lower serum levels, the following gene/genotypes have been observed: KNG1 rs11924390 T/T; ANKH rs2454873 G/G; NPFFR2 rs4129733 T/G; SH2B1 rs4788102 G/A; RAP1A rs494453 T/T and CRHBP rs7728378 T/C. These genes were previously associated with the risk of osteoporosis, type 2 diabetes, obesity, or stress response, but interestingly, most of them are independent of the canonical pathways of vitamin D synthesis or metabolism [88,89].

As shown in the example of vitamin D, the level of a given compound in the body, as well as the effectiveness of its supplementation, depends on many factors, such as the dose of the supplement, but also individual factors, such as genetic polymorphisms or BMI. Methods used in the field of omics are beneficially used here in assessing the effect of a given supplement on transcripts, metabolites, or the proteome and, in finding a possible reason for the lack of the assumed response of the organism to the administered compounds. Some of the most significant publications describing the use of omics techniques in the study of vitamin D are listed in Table 1.

#### 4.1.2. Vitamin E

Vitamin E is a term for a group of eight fat-soluble compounds, of which α-tocopherol has the highest biological activity. It functions mainly as an antioxidant that protects cell membranes from oxidative damage and along with vitamin A plays a role in vision [90]. Vitamin E deficiency, for which the symptoms are mostly neurologic, rarely occurs in humans and only as a result of abnormalities in dietary fat absorption or metabolism, for instance, due to mutation in genes coding alpha-tocopherol transfer protein (α-TPP) [91].

For decades, vitamin E supplementation has been tested for cardiovascular disease or cancer, mostly prostate cancer and prevention, with mixed results. Some trials show that a daily dose of 50 IU alpha-tocopheryl acetate (ATA) can reduce prostate cancer incidence by about 40%, while on the other hand, a daily dosage of 400 IU followed for 7–12 years, increased prostate cancer incidence by 17% [92,93]. The study conducted by Huang et al. aimed to measure the serum biochemical changes in men receiving 50 IU ATA or 400 IU ATA to gain biochemical insight into vitamin E dosage-related effects potentially relevant to prostate cancer findings [94]. Fasting serums were collected from participants of two distinct trials—VEAPS (400 IU) (clinicaltrials.gov (NCT00114387)) and ATBC (50 IU) (clinicaltrials.gov (NCT00342992)). With the use of a high-resolution accurate mass (HRAM) platform of ultrahigh-performance liquid chromatography/tandem mass spectroscopy (LC-MS/MS), baseline and follow-up serum metabolites were measured. After excluding unknown compounds, 974 metabolites, classified into eight chemical classes (amino acids, carbohydrates, cofactors and vitamins, energy metabolites, lipids, nucleotides, peptides, or xenobiotics), were included in the final analysis. Testing the biochemical effects of supplementation with either 50 IU or 400 IU ATA daily showed that in addition to the anticipated significant increase in alpha-CEHC sulfate and alpha-tocopherol in both trials, a novel C22 lactone sulfate compound was significantly decreased only in the high-dose VEAPS trial. In addition, most of the androgenic steroid metabolites that directly correlated with serum C22 lactone sulfate were significantly reduced by ATA supplementation only in VEAPS. These investigations point to a direct impact of high-dose vitamin E supplementation on this lactone-containing metabolite that correlated with androgen metabolites potentially relevant to elevated prostate cancer incidence [94].

Clinical trials have generally failed to demonstrate reduced cardiovascular risk with vitamin E supplementation, but as reactive oxygens species may contribute to the progression of diabetes complications, vitamin E antioxidant functions may be exceptionally beneficial for individuals with diabetes or Haptoglobin (Hp) 2-2 genotype (polymorphism in *Hp* gene that binds to free hemoglobin and is HDL-related). Costacou et al. conducted a crossover study to evaluate the effects of α-tocopherol supplementation on HDL (high-density lipoprotein) function in type 1 diabetes, stratifying by Hp genotype. Participants within each Hp genotype were randomly allocated to eight weeks of daily 400 IU α-tocopherol acetate or placebo. Collected plasma samples were analyzed with the use of NMR lipoprotein subfraction analysis. Particle concentrations of lipoproteins of different sizes were calculated from the measured amplitudes of their spectroscopically distinct lipid-methyl group. Findings suggest that α-tocopherol may improve HDL-mediated cholesterol efflux, a dysfunction that may be at least partly responsible for the observed cardiovascular susceptibility in Hp 2-2 carriers in type 1 diabetes. The results also suggest that vitamin E may reduce LDL (low-density lipoprotein) particle concentration in this Hp subgroup, with no benefit; indeed somewhat adverse changes in lipoprotein subfractions and lipid peroxides were observed in Hp 1 allele carriers. Therefore, these findings provide some support for the hypothesis that α-tocopherol supplementation, while not beneficial for the general or diabetes population overall, may benefit those most susceptible to cardiovascular disease, i.e., individuals with diabetes who carry the Hp 2-2 genotype [95].

#### 4.1.3. Vitamin A

Vitamin A is a term for a family of retinoids (retinol, retinaldehyde, retinoic acid), with provitamin A (carotenoids, most prevalent is B-carotene) as a precursor. This vitamin is an essential fat-soluble micronutrient obtained from the diet, which absorption can be impaired by zinc deficiency and alcohol consumption [96]. The deficiency of this micronutrient is considered one of the most prevalent deficiencies worldwide, mainly affecting children (globally about 30% of children under the age of 5) in developing countries. Vitamin A deficiency can lead to blindness and cutaneous aberrations, can make a person prone to infections, and can increase the risk of cancer development [97]. As dangerous as deficiency is, the excessive intake of vitamin A can lead to neurological side effects, osteoporosis, and abnormalities during embryogenesis [98,99,100].

The multiplicity of retinoids bioactivities is largely due to their ability to regulate the expression of targeted genes, primarily through nuclear retinoid receptors [101,102]. To determine changes in gene expression levels due to vitamin A supplementation, Wang et al. conducted a study to evaluate the metabolomic changes in RAW264.7 cells. Cells were treated with retinoid at an IC50 dose. RAW264.7 cells showed significant changes in protein biosynthesis, urea cycle, arginine and proline metabolism, malate-aspartate shuttle, alanine metabolism, and cellular respiration after retinol treatment IC50 dose (140 μM), indicating that retinol affects the cellular physiological functions via different metabolic pathways. With the use of ^1^H-NMR 22 metabolites, such as amino acids, sugars, organic acids, glutathione, glycerin, and creatinine were identified as changed upon treatment. The cells also showed significantly increased levels of oxidative stress, IL-6 (interleukin 6) and TNF-α (tumor necrosis factor α), likely because of the mitochondrial dysfunction and lipid peroxidation caused by retinol treatment [103]. Therefore, it is possible to identify which metabolic changes occur under the influence of a given supplement and determine its potential toxicity using NMR techniques.

### 4.2. Plant Extracts

#### 4.2.1. Resveratrol

Resveratrol (3,5,4′-trihydroxystilbene, RSV) is a small, natural polyphenol found mainly in the skin of red grapes, berries, tea, nuts, and blueberries and in dark chocolate. RSV exists in two isomeric forms, but the “trans” form is predominant and is used as a food supplement or in the cosmetic industry. In recent years there has been a growing interest in the potential health benefits of resveratrol supplementation, which is reported to have anti-inflammatory, antioxidant, anti-hyperlipidemic, immune-modulator, anti-carcinogenic, cardio- and neuroprotective, and anti-aging effects. However, the potential effects of resveratrol were recognized through in vitro, ex vivo, and animal studies while human clinical studies conducted so far did not confirm many of the above actions of RSV, and some even suggested toxicity [104,105].

There are a few studies investigating the influence of resveratrol on the transcriptome or proteome [106,107,108,109,110], but in order to determine the effect of a substance in vivo and in clinical trials, it is also necessary to investigate the distribution and level of the compound in the blood and other biological fluids, in the tissues where the compound is metabolized but, in some cases, especially when the compound has a short biological half-life or poor aqueous solubility, such as resveratrol, this can be a challenge. In such cases, the LC-MS/MS method may be appropriate, as was shown with resveratrol, for which the distribution in the mouse plasma and brain was successfully determined with the use of this method, which supports pharmacokinetic studies [111].

#### 4.2.2. Green Tea

Tea is the second most consumed beverage around the world, after water, and although there are many categories and varieties of tea, green tea is gaining popularity not only as a relaxing drink but also as having many health benefits. Tea is produced from the leaves, buds, or stems of the plant *Camellia sinensis*. Depending on the level of antioxidants present and the degree of fermentation, there are three major forms of tea—black tea, oolong tea, and green tea. Green tea is categorized as nonfermented, and mainly (24–36% in dry weight) consists of catechins (polyphenols). Besides polyphenols, tea consists of caffeine, flavonoids, lignin, amino acids, organic acid, and chlorophyll. Numerous findings suggest that green tea may impact the cardiovascular system’s functioning, reduce body mass, and even decrease the risk of neurodegenerative diseases or cancer. In vitro studies have shown that green tea polyphenols (GTP), mostly (-)-epigallocatechin-3-gallate (EGCG) are antioxidants free-radical scavengers and can induce autophagy. As shown with the use of omics analyses of the microbiome, such as LC-MS/MS and single-cell transcriptomics, tea polyphenols can also regulate the circadian rhythm by affecting intestinal flora and related metabolites and can regulate circadian gene expression [112,113]. Due to the effects mentioned above, green tea extract (GTE) supplementation has become common, despite no published data on the effects of long-term GTE consumption and studies that showed that EGCG may induce cytotoxicity to liver cells [114,115,116,117].

Green tea may have an anti-obesity effect, and to assess the underlying molecular mechanisms, next-generation sequencing can be used. Zang et al. using zebrafish larva and adult obesity models showed that the daily administration of GTE reduced visceral adipose tissue volume and based on the RNA-sequencing of liver tissue, this may be due to the activation of Wnt/β-catenin and adenosine monophosphate-activated protein kinase pathways [118]. A similar study conducted on an obesogenic mice model proved that green tea contributes to systemic metabolic homeostasis by regulating the expression of specific genes involved in BCAA (branched chain amino acids) degradation and lipid and glucose metabolism [119].

#### 4.2.3. Ginseng

Traditionally used in Chinese medicine ginseng is the most widely consumed herb globally [120]. The main phytochemical constituents in ginseng are triterpene saponins (the ginsenosides), which occur in different proportions depending on the ginseng species. Fresh ginseng root is used in food recipes, while ginseng extract or powder are used as supplements due to ginseng’s reputation as a panacea for various physical and mental issues. Ginseng is believed to help manage stress, memory loss, or fatigue and can help treat diabetes, respiratory infections, cardiovascular disease, inflammatory disorders, and even breast or prostate cancer. Some studies assessing the effect of ginseng on the transcriptome, proteome, or metabolome have already been carried out [121,122,123,124,125]. However, meta-analysis and clinical trials did not confirm the positive influence of ginseng on overall health or in treating mentioned conditions [115,126,127]. Due to no substantial evidence, the US Food and Drug Administration (FDA) did not approve ginseng as a drug [128]. Nonetheless, ginseng products are commonly marketed as a dietary supplement but should be taken with extreme caution as when taken in excessive use, it may have a variety of side effects and interactions with drugs.

On the packaging of a dietary supplement, there is often only general information about its ingredients. As in the case of ginseng preparations, usually, only the name of the species and its percentage are given. Meanwhile, depending on the variety, age of the plant and the processing method, the content of primary metabolites varies [129,130]. For instance, there are four processed P. ginseng products, white ginseng, tae-geuk ginseng, red ginseng, and black ginseng which differ in the sugar content that is shown with the use of high-resolution magic angle spinning (HR-MAS) NMR-based metabolomics [130]. Additionally, each cultivar has its characteristic profile of primary and secondary metabolites, which was shown in the example of seven cultivars (Chunpoong, Chungsun, Kumpoong, Yunpoong, Gopoong, Sunwon, and Sunun) that were analyzed using ultra-performance liquid chromatography-quadrupole-time-of-flight mass spectrometry (UPLC-QTOF/MS) and HR-MAS NMR spectroscopy, which allowed the study to determine the differences in the level of ginsenosides and amino-acids [131].

#### 4.2.4. Curcumin

Curcumin is another example of an ingredient used daily as a food product that has become a dietary supplement under the influence of reports of a broad, beneficial effect on health. Curcumin (CUR; polyphenol) is one of the main compounds found in the rhizome of turmeric (*Curcuma longa),* a plant that is usually used as a spice used in the preparation of curries in Asian countries or as a coloring agent (vivid orange-yellow color) in a variety of industries. In Ayurvedic medicine, turmeric is also known to be helpful in respiratory problems, dermatological disorders, or wound healing. In recent decades, curcumin attracted the attention of scientists due to its therapeutic potential as anti-diabetic, anti-inflammatory, antioxidant, anti-cancer, anti-aging, and even as a treatment component in chronic diseases such as Alzheimer’s disease, Parkinson’s disease, rheumatoid arthritis, migraines, and many more. Furthermore, while there are quite a lot of studies on the effect of curcumin on transcriptome or proteome [132,133,134,135,136,137], including ones conducted with omic methods, unfortunately, this influence has not been confirmed in any of the official, reliable clinical trials. One of many concerns, with the use of curcumin extract as a potential drug, is its instability, poor solubility, and low bioavailability [138,139,140,141,142,143,144].

In connection with reports of the influence of curcumin on certain lung diseases, such as asthma, lung cancer, or pulmonary fibrosis, researchers from Taiwan hypothesized that this compound might have a positive effect on the course of chronic and progressive lung disease, idiopathic pulmonary fibrosis (IPF), characterized by fibrosing interstitial pneumonitis. Some in vitro and in vivo studies showed that curcumin might promote apoptosis, inhibit differentiation and enhance antioxidant mechanisms in fibroblasts, but the effect of curcumin on modulating gene expression profiles in those cells was not investigated. Therefore, using next-generation sequencing and bioinformatic analyses, Chang et al. explored the effects of curcumin on mRNA and microRNA changes in IPF fibroblasts. Thanks to the use of NGS it was possible to identify several dozen down- and up-regulated protein-coding genes and microRNAs in curcumin-treated IPF fibroblasts, which are involved in the pathways responsible for the suppression of cell cycle progression. They found that curcumin might decrease the level of hsa-miR-6724-5p, leading to increased KLF10 expression and resulting in cell cycle arrest in fibroblasts, which supports the potential role of this compound in the treatment of IPF.

## 5. Conclusions

Omics are areas of science that are considered essential to the holistic research and development of many vital issues, such as personalized medicine and the efficient and safe production of specific drugs. Techniques from the domain of omics should also be widely used in the study of dietary supplements. As shown in the examples in the review, the most commonly used omics techniques, such as NGS, LC-MS, and NMR, help investigate the molecular-level effect of a supplement in specific cases and its safe use. Variants of NGS techniques are most useful in establishing new potential effects of supplementation and in identifying signaling pathways and biological processes that are actually altered by the component. In addition, NGS allows the identification of groups of people, for example, with various polymorphisms or specific syndromes, which may be predisposed to a deficiency of a given compound and in which supplementation would have an actual, commensurate effect of bringing the organism to homeostasis. LC-MS techniques are most helpful in assessing the effects of various doses of the supplemented compound on metabolites and proteins. They are also used to measure the serum content of a given compound that would reflect the supplemented doses well. LC-MS techniques can also be used in studying the distribution and level of metabolites in compounds. Moreover, the variants of NMR techniques should be used to study metabolic changes and metabolic action pathways. They are also helpful in studying the potential toxicity of a compound. NMR can establish the exact composition of the compound, its sugar content, and primary and secondary metabolites. Moreover, those methods can be used to determine what conditions or individual characteristics may be affecting the effectiveness of supplementation. Therefore, by using these modern techniques and their combinations, the possibility of thoroughly examining the ingredients of dietary supplements and determining for whom such supplementation may be useful or necessary, and at the same time safe, become widely available.

## Figures and Tables

**Table 1:** List of publications describing the use of omics techniques in the study of vitamin D.

First Author	Year of Publishing	Purpose	Used Method
Carlberg	2019	Modulation of the epigenome of immune cells by vitamin D status	ChIP-seq, FAIRE-seq
Carlberg	2018	Response of the human epigenome to vitamin D supplementation	FAIRE-seq
Seuter	2016	Molecular evaluation of vitamin D responsiveness of adults	FAIRE-seq
Wilfinger	2014	Utility of primary vitamin D receptor target genes as biomarkers for the vitamin D3 status	ChIP-seq, FAIRE-seq
Saksa	2015	Dissecting high from low responders for the vitamin D3 supplementation	ChIP-seq
Tuoresmaki	2014	Localizations of vitamin D receptors in genome	ChIP-seq
Lu	2018	Review connecting genomic effects of vitamin D on immune cells with multiple sclerosis	ChIP-seq
Benson	2017	Method analysis: Designing of a custom next generation sequencing panel for vitamin D associated genes	ION AmpliSeq, ION S5 Cl system
Cheng	2020	Effects of vitamin D on the immunomodulation of head kidney in yellow catfish	NGS
Zumaraga	2021	NGS of the entire vitamin D receptor gene in order to investigate polymorphisms and correlation with vitamin D deficiency	targeted NGS
Zumaraga	2016	NGS in order to detect genetic polymorphisms correlated with vitamin D deficiency	targeted NGS
Silva	2016	Studying changes in gene expression in CaCo2 cells upon vitamin D treatment	NGS
Hänninen	2020	Influence of vitamin D supplementation on sNfL (serum neurofilament light chain), that are promising biomarkers of MS activity	MRI
McClorry	2019	Impact of BMI and serum fructose on effectiveness of vitamin D supplementation on children	NMR
Bislev	2020	Investigation of cardiovascular and musculoskeletal health upon daily supplementation of vitamin D	NMR
Rana	2014	Effects of vitamin D supplementation on muscle energy phospho-metabolites	P magnetic resonance spectroscopy
Sheedy	2014	Analysis of human urinary metabolome in response to calcium-vitamin D3 supplementation	H-NMR
Ponda	2012	Inluence of vitamin D oral supplementation on the lipid profile	NMR-based lipid fractions
Chen	2015	Association of consumption of vitamin D2 enhanced mushroom with improved bone health in mice	NMR
